# The quality of life of people with epilepsy at a tertiary referral centre in Malaysia

**DOI:** 10.1186/1477-7525-11-143

**Published:** 2013-08-23

**Authors:** Bachok Norsa’adah, Jiman Zainab, Aishah Knight

**Affiliations:** 1 Unit of Biostatistics and Research Methodology, School of Medical Sciences, Universiti Sains Malaysia, Kubang Kerian, Kelantan, Malaysia; 2 School of Health Sciences, Universiti Sains Malaysia, Kubang Kerian, Kelantan, Malaysia; 3 Advanced Medical & Dental Institute, Universiti Sains Malaysia, Kepala Batas, Penang, Malaysia

**Keywords:** Epilepsy, Quality of life, QOLIE-31, Frequency of seizures, Malaysia

## Abstract

**Background:**

Epilepsy, a chronic disorder of brain characterised by a predisposition to generate epileptic seizures, has an effect on the psychosocial well-being of sufferers. Measuring the quality of life (QOL) of people with epilepsy (PWE) is increasingly recognized as an important component of clinical management. QOL measures differ between countries and there is limited information regarding PWE in Malaysia. The aim of this study was to determine the health related QOL and its relationship with the presence of seizures in PWE at a Malaysian tertiary referral center.

**Methods:**

A total of 106 adults with epilepsy attending the weekly neurology clinic of Universiti Sains Malaysia Hospital were interviewed in this cross-sectional study. The QOL was measured using a validated Malay translated version of the Quality of Life in Epilepsy Scale-31 (QOLIE-31). Analysis of covariance was used for data analysis.

**Results:**

The mean age was 31.8 years old (standard deviation (SD) 11.0) and 56.6% were females. The mean total score of QOLIE-31was 68.9 (SD 15.9). The highest subscale score was the medication effects with a mean of 79.4 (SD 28.5) and the lowest was seizure worry with 47.5 (SD 25.0). Respondents who had one or more seizures in the past four weeks had significantly lower mean score of QOL [63.4, 95% CI: 59.2, 67.5] than those who had no seizure [mean score 73.5, 95% CI: 69.3, 77.9] after adjusting for age, sex, treatment, duration and age at onset of epilepsy [F test =11.215, p = 0.001, R^2^ = 0.159]. All the sub-scales of QOL showed significant moderate correlation with the frequency of seizures except for cognitive functioning.

**Conclusions:**

Worrying about seizure had the major contribution on QOL, while medication effect had the least. This study confirms the importance of seizure control for a better QOL in Malaysian patients with epilepsy.

## Introduction

Epilepsy is a common serious neurological disorder, caused by abnormalities in the electrical activity of the brain [1]. The diagnosis of epilepsy requires two elements: a history of at least one seizure and evidence of an enduring alteration in the brain activity [2]. It has been estimated that more than 200 thousand people in Malaysia suffer from epilepsy [3] but there is a lack of data on the present prevalence of epilepsy in Malaysia [4].

During the last few decades, greater attention has been given to the quality of life (QOL) of people with epilepsy (PWE). They have been shown to report a poorer QOL compared to the general population because they are more likely to have poor self-esteem and a high level of anxiety and depression [5]. Social stigma and discrimination of PWE occur in both developed and developing countries [5,6]. In many countries, this disorder is still shrouded in secrecy and patients prefer not to reveal or discuss their condition [7]. In Malaysia, a survey to assess public awareness and knowledge reported that even though there was a high awareness of the condition, many of the 839 respondents had negative attitudes and poor knowledge on epilepsy [8]. Previous studies have reported that QOL of PWE is also influenced by both clinical characteristics (such as frequency, type or presence of seizures, age of onset and treatment) and socio-demographic variables (such as sex, marital status, education and employment) [6,9-11].

Currently, PWE are high users of health care resources, including medications, emergency care services, hospitalizations, psychological or social services, nursing services and other consultations with health professionals [12,13]. Understanding the unique and complex impact of epilepsy on a person’s QOL is also increasingly recognized as an important component of clinical care [14] and research in this area will identify factors affecting QOL and may lead to strategies that improve the management of PWE.

Very few studies have been carried out on epilepsy and none have been published on measurement of QOL in PWE in Malaysia. Different countries have dissimilarities of beliefs, culture and socio-economic factors which in turn can affect QOL measures, thus findings from other countries, may not be relevant to the local situation. This study was conducted to determine the level of health related QOL of PWE under follow-up in Hospital Universiti Sains Malaysia (HUSM).

## Methods

HUSM is a teaching hospital located in north-eastern peninsular Malaysia. It is a tertiary referral center for the area and serves a population that has a lower socio-economic status compared to the population in the capital city Kuala Lumpur located on the west coast of peninsular Malaysia. Annually there are about 800 consultations by PWE at the neurology clinic and the interval for routine follow-up is approximately three months.

This was a cross sectional study in design. Respondents were adults aged at least 18 years old with a diagnosis of epilepsy who were attending follow-up at the neurology clinic during the study period. We included all types of epilepsy that had been diagnosed for at least a year; respondents also had to be seizure free for 24 hours and consented to participate in the study. We excluded people with psychotic disorders, severe mental retardation, strokes, head injuries, brain tumors and patients who have had recent brain surgery.

Questionnaires were developed to collect socio-demographic data (age, sex, marital status, employment status, educational level and monthly household income) and clinical aspects of epilepsy (frequency, type and presence of seizures, the age of onset, duration of epilepsy, etiology and medication). Seizures frequency was defined as the number of seizures occurring in the four weeks prior to the interview. We also collected data on co-morbidity of other chronic diseases such as diabetes, hypertension and asthma.

QOLIE-31was used for collecting data on health related QOL with the permission of the RAND Corporation. QOLIE-31consists of seven subscales which are seizure worry (five items), emotional well-being (five items), energy / fatigue (four items), cognitive functioning (six items), medication effects (three items), social functioning (five items), overall QOL (two items) and one item of overall health [15]. The responses used Likert rating scales which were later transformed into linear scales that ranged between 0 and 100. A higher score indicates better quality of life.

The translation of QOLIE-31into Malay version was done by two individuals who are fluent in both the English and Malay languages. The new Malay version of QOLIE-31was back-translated into English to ensure that the meaning and comprehension of the original version was retained. The Malay version of QOLIE-31was also checked for both the accuracy and meaning of the translated versions before being finalized. A pretest was conducted on 20 PWE to ensure that the translated questionnaire was easy to understand. The overall Cronbach’s alpha score was 0.697. The scoring and weightage of each item in the QOLIE-31 was obtained from the guidelines in the QOLIE-31 manual [15]. The total scores of subscales and total QOL are presented in the results section.

The Research and Ethics Committee of Universiti Sains Malaysia approved this study and all data pertaining to the study were kept confidential. Written informed consent was obtained from the patients for the participation in this research and publication of the findings. Data were analyzed using the Statistical Package of Social Sciences (SPSS) version 12.0. Descriptive statistics were expressed as mean (standard deviation (SD)) and frequency (percentage) as appropriate. The independent t- test or one-way analysis of variance (ANOVA) was used to compare means of QOL scores between groups. Analysis of covariance (ANCOVA) was used to compare the means of QOL scores between the presence of seizures with the adjustment of age, sex, treatment, duration and age of onset. Correlation coefficient was used to measure the relationship between frequency of seizures and total score of QOLIE-31. The level of significance was set at p value < 0.05.

## Results

Out of a total of 120 PWE approached, 106 fulfilled all the inclusion and exclusion criteria and were included in the analysis. The age of respondents ranged from 18 to 62 years old with the mean (SD) of 31.8 (11.0) years. More than half were female (56.6%). The range of seizures frequency in the past 4 weeks was 0–8, with mean (SD) of 1.09 (1.52). The mean (SD) age at onset of epilepsy and duration of epilepsy were 17.2 (13.5) and 14.1 (9.0) years respectively.

Table 1 shows the mean and SD scores of QOLIE-31 subscales. The mean (SD) total score of QOLIE-31 was 68.9 (15.9). The highest mean (SD) score was the medication effects, 79.4 (28.5) and the lowest was seizure worry subscale, 47.5 (25.0). One-way ANOVA showed significant differences in the mean score of sub-scales of QOLIE-31 (p < 0.001). There were significant differences between subscales scores in post-hoc tests. The total score of seizure worry and medication effects were significantly different with all other subscales in QOLIE-31 (p < 0.001). The table also shows the results of the Cronbach's Alpha for each sub-scale.

**Table 1:** Total score of QOLIE-31 sub-scales

**Subscales of QOLIE-31**	**Mean (SD) score**	**Cronbach's Alpha**
Seizure worry	47.5 (25.0)	0.766
Overall quality of life	72.8 (15.0)	0.614
Emotional well-being	70.1 (18.8)	0.692
Energy/fatigue	64.9 (19.6)	0.679
Cognitive functioning	70.2 (21.1)	0.779
Medication effects	79.4 (28.5)	0.796
Social functioning	72.9 (25.4)	0.819
Subjective overall health (Item 31 – visual analogue scale)	75.9 (16.6)	
Total Score	68.9 (15.9)	0.697

One-way ANOVA, F test =21.376, p-value <0.001.

Tables 2 and 3 show the different total QOL scores for groups with different socio-demographic and clinical characteristics. There were no significant differences in the mean of total QOL scores between groups for socio-demographic and clinical characteristics except frequency of seizures. ANCOVA analysis confirmed a significant mean difference in the total score of QOLIE-31with the occurrence of seizures in the past four weeks [F test =11.215, p = 0.001, R^2^ = 0.159], after adjusting age, sex, treatment, duration and age at onset of epilepsy. Respondents who had no seizure in the past four weeks had higher QOL [mean score 73.5, 95% CI: 69.3, 77.9] than those had seizure [mean score 63.4, 95% CI: 59.2, 67.5]. Table 4 shows the correlation between seizures frequency and total score and sub-scales of QOLIE-31. All the sub-scales showed significant moderate correlation with the seizures frequency except cognitive functioning which showed weak correlation.

**Table 2:** Differences of QOL score for socio-demographic characteristics of the respondents

	**N (%)**	**Total QOL score**	**p-value**
		**Mean (SD)**	
Age (years)			0.597^a^
18-25	39 (36.8)	68.7 (15.9)	
26-35	31 (29.2)	67.0 (13.6)	
36-62	36 (34.0)	70.9 (18.0)	
Sex			0.201^b^
Male	46 (43.4)	66.7 (15.7)	
Female	60 (56.6)	70.7 (16.0)	
Marital status			0.591^b^
Single	64 (60.4)	68.3 (14.9)	
Ever married	42 (39.6)	70.0 (17.5)	
Employment status			0.985^b^
Employed	39 (36.8)	69.0 (17.5)	
Unemployed	67 (63.2)	68.9 (15.1)	
Educational level			0.641^a^
Lower secondary or lower	37 (34.9)	66.9 (17.8)	
High secondary	48 (45.3)	69.8 (12.1)	
Higher than high secondary	21 (19.8)	70.4 (20.3)	
Monthly household income (RM)			0.330^a^
<500	21 (19.8)	64.3 (16.7)	
500-1,000	61 (57.5)	70.2 (15.7)	
>1,000	24 (22.7)	69.7 (15.7)	

^a^One-way ANOVA, ^b^independent t test.

**Table 3:** Differences of QOL score for clinical characteristics of the respondents

	**N (%)**	**Total QOL score**	**p-value**
		**Mean (SD)**	
Age of onset (year)			0.096^a^
<10	30 (28.3)	69.6 (16.0)	
10-25	50 (47.2)	65.0 (16.4)	
>25	26 (24.5)	74.1 (13.8)	
Type of seizure			0.982^b^
Partial	27 (25.5)	69.0 (17.0)	
Generalised	79 (74.5)	68.9 (15.7)	
Duration of epilepsy (year)			0.379^a^
<5	15 (14.2)	73.0 (14.0)	
5-20	69 (65.1)	69.1 (15.0)	
>20	22 (20.7)	65.6 (19.6)	
Treatment			0.131^b^
Monotherapy	61 (57.5)	70.9 (16.0)	
Polytherapy	45 (42.5)	66.2 (15.6)	
Co-morbidity			0.871^b^
No	87 (82.1)	69.2 (15.8)	
Yes	19 (17.9)	67.7 (17.1)	
Family history			0.891^b^
No	94 (88.7)	68.9 (16.0)	
Yes	12 (11.3)	69.5 (16.1)	
Etiology			0.811^b^
Idiopathic	55 (51.9)	69.3 (16.5)	
Other factors	51 (48.1)	68.5 (15.4)	
Presence of seizure in past 4 weeks			<0.001^b^
Absent	54 (50.9)	74.3 (14.1)	
Present	52 (49.1)	63.3 (15.9)	

^a^One-way ANOVA, ^b^independent t test.

**Table 4:** Correlation between frequency of seizures and total score of QOLIE-31 sub-scales

**Subscales of QOLIE-31**	**Correlation coefficient**	**P value**
Seizure worry	0.431	<0.001
Overall functioning	0.368	<0.001
Emotional well-being	0.382	<0.001
Energy/fatigue	0.382	<0.001
Cognitive functioning	0.190	0.051
Medication effects	0.390	<0.001
Social functioning	0.359	<0.001
Total Score	0.436	<0.001

Table 5 shows the distribution of anti-epileptic drugs used by the respondents. More than half were on monotherapy and the most frequently used medications were sodium valproate, followed by carbamazepine and lamotrigine.

**Table 5:** Anti-epileptic drugs used by the respondents

**Anti-epileptic drug**	**No (%)**
Sodium valproate	56 (52.8)
Carbamazepine	31 (29.2)
Lamotrigine	28 (26.4)
Levetiracetam	18 (17.0)
Phenytoin	16 (15.1)
Clonazepam	5 (4.7)
Topiramate	4 (3.8)
Phenobarbitone	1 (0.9)
One type	61 (57.6)
Two types	38 (35.8)
Three types	7 (6.6)

## Discussion

The mean total score of QOLIE-31 in our study [68.9 (SD15.9)] was higher than other studies conducted in Moscow [42.13 (SD 4.14)] [6], Australia [52.9 (SD 23.1) [16], Togo [49.5 (SD 14.4)] and Benin, Africa [52.1 (33.4)] [17]. Malaysia is a country which has specialist-physician run services for PWE [18] and this may have contributed to a better standard of medical care, perhaps resulting in a better HRQOL score. It must be noted, however, that even though the studies cited had used the QOLIE-31 questionnaire (different translations) or QOLIE −89 questionnaire (the other questionnaire for measuring QOL in epilepsy patients from the RAND corporation), different study methodologies with different inclusion and exclusion criteria could also account for the different scores.The pattern of scores of QOLIE-31 subscales of our study was similar to the studies conducted in Africa [17] with medication effects having the highest score while seizure worry had the lowest. In Australia where the respondents studied were generally older, the social function subscale was the highest and energy/fatigue was the lowest score [16]. This difference in patterns is likely due to differences in sociocultural or clinical factors and it has been shown that country of origin has an effect on quality of life scores [19]. Although direct comparison between QOL scores of different countries is not advisable, finding a QOL score that is not too dissimilar to other countries is somewhat reassuring.

The subscales of the translated QOLIE-31 questionnaire showed satisfactory (more than 0.7) Cronbach’s Alpha scores were seizure worry, cognitive functioning, medication effects and social functioning. Emotional functioning and energy/fatigue had scores that were close to satisfactory. Only overall functioning, which had only two items, scored 0.6. These satisfactory scores for the items in the subscales may attest to the validity of the translated questionnaire.

Nearly half of our respondents had at least one episode of seizure in the past four weeks, which is a poor result when compared to a study in Iran, the Gulf and Near East that reported only 25% of epilepsy respondents having one or more seizures per month [5]. Being a tertiary centre, it is possible that our respondents were more complicated cases compared to the respondents in that study who were sourced from hospital outpatients.

Only seizures frequency was found to have a significant effect on the QOL score among all the clinical characteristics that were measured. No socio-demographic characteristic was found to be of significance. There were significant weak and moderate correlations between seizures frequency and all the QOL sub-scales except for cognitive functioning (P = 0.051). The correlation strength between scores of subscales and seizures frequency in our study were stronger compared to a study in Moscow [6] which also found a significant relationship between seizures frequency and QOL. In general, the literature supports the finding of our study in which people with frequent seizures had significantly poorer HRQOL than those with infrequent or no seizures [10,11,17,19-22]. Baker et al. reported that seizures frequency was the most important clinical predictor of psycho-social dysfunction and emotional maladjustment in PWE [9]. McLaughlin et al. concluded that even infrequent seizure might impair health related QOL in older PWE [16]. People with uncontrolled epileptic seizures will always be in the uncomfortable position of not knowing when the next seizure will occur and may take precautions and impose restrictions to avoid of having seizures at inappropriate times, public places or social events. They can be restricted from driving, and may be denied job and career opportunities which can translate into a lower QOL. The threat of recurrent seizures and the fear of social embarrassment and rejection can be lifelong concerns for PWE. There is a hazard of injury during a seizure that may also affect their QOL. Our study also showed that seizure worry was significantly the lowest score among all the sub-scales of QOLIE-31.

Frequency of seizures has been related to fear and misunderstanding which results in the social stigma and discrimination surrounding epilepsy. A study in Zambia reported that many PWE were feared and rejected by families or communities [23].Cultural beliefs influence attitudes and actions of the community towards PWE [8,24] and high rates of anxiety, depression, and low self-esteem have been measured in PWE, thus adversely affecting quality of life [25]. In Malaysia, a study of university students reported that many students could not differentiate between mental illness and epilepsy and it was common to use many insanity related terms to describe PWE [26]. It seems that even in a setting of higher education, there is a lack of understanding of epilepsy and greater efforts are needed to raise awareness and understanding about the condition for the general public by the local authorities.

Longer duration of epilepsy has been reported as a predictor for poor QOL [9,21]. The longer duration of epilepsy may be related to greater complications and disabilities. Our study found no significant association between duration of epilepsy and QOL, although there was a trend of lower scores of QOL in those with longer duration of epilepsy. The number of respondents in our study was quite small in number compared to the study by Baker et al. [9] and there were more women in our sample compared to the study by Djibuti et al. [21]. There were more respondents with generalized than partial seizures in our study. The type of seizure has not been shown to be an important predictor factor of QOL [9,27] and this was also found in our study.

Less seizures frequency has been related to a greater likelihood of being employed [28]. More than half of our respondents reported being unemployed. The reasons for unemployment are multi-factorial, and are related to the state of seizure control, the age of onset and duration of illness, the type of medication, severity and frequency of seizures [20,28]. It is likely that a person with epilepsy diagnosed in childhood would have many schooling hours disrupted if the frequency of seizures were uncontrolled and this could also lead to low educational achievement and thus a lower employability potential. Occupational related stress such as assignment deadlines and criticisms by supervisors may trigger seizures. Furthermore, a seizure itself may cause accidents and injuries thus preventing sufferers from driving and depriving many types of employment opportunities. Employers are unlikely to employ a person with epilepsy if they are experiencing seizures that interfere with job performance.

Our study did not find any significant differences in the QOLIE-31 and the presence of co-morbidities as supported by Alanis-Guevara et al. [10]. Psychiatric diseases such as depression and mood disorders have been reported to be common among PWE and are important determinants of QOL [22,29]. The presence of co-morbidities in our study was confirmed only by reviewing the general medical records, which was one of our study limitations. We did not use specific tools to measure psychiatric co-morbidity as was done in studies that identified such co-morbidities as significant predictors of QOL.

Seizure remission is possible with appropriate type and dose of long term antiepileptic drugs (AEDs). PWE who were treated with AEDs had significantly better QOL than the untreated people [16]. Monotherapy has been recognized to be able to control seizures adequately and polytherapy is only necessary in the minority of people with more severe seizures. A study in Malaysia reported that the percentage of people that received monotherapy was nearly double than those with polytherapy [3]. In our study, 57.5% was on a single drug therapy, 35.8% on two types, 6.6% on three and none on four types of AED. In comparison, an Iranian study reported that 61% of their respondents were receiving monotherapy, 29% were taking two, 7% were taking three and 1% were taking four of five AEDs [5]. It has been reported that there was no association between polytherapy with QOLIE-31 [6,10,27] and this is also confirmed in our study, respondents who were on polytherapy did not have poorer HRQOL compared to those on monotherapy. There is evidence that suggests PWE who are seizure free without significant adverse drug effects have QOL measures as good as individuals without epilepsy [19].

Our study used a Malay version of QOLIE-31 that had a satisfactory reliability test. QOLIE-31 is an accurate, specific and complete measurement for HRQOL among PWE [15,21]. It has been designed as a brief format to assess health related QOL among PWE; and is an easily scored questionnaire that has been tested in many languages and populations [15]. It can be used as a tool for clinicians and researchers to determine the magnitude of the effect of interventions on QOL. We have shown that frequency of seizures is a predictor of QOLIE-31 scores, therefore if QOL measures are to be used to assess between different treatment regimens in Malaysia, comparisons should be done with patients stratified into those with controlled and uncontrolled seizures

This study aimed to assess the HRQOL of PWE and only sourced the data from the neurology clinic of HUSM, thus, the results may not be generalized to PWE in Malaysia. All the respondents were on follow up at a tertiary level academic hospital, and may have been refractory to first-line treatment or having other complications that might affect their QOL compared to PWE who were on follow up in primary care centers.

Further research on the HRQOL among PWE in Malaysia is still needed. Comparison of HRQOL between PWE and controls as well as to identify the psychiatric co-morbid conditions that may affect QOL scores could be very useful as many psychiatric co-morbid conditions can be managed with non-drug regimens.

Epilepsy often begins at a young age and affects young people in their most productive years of their lives and can affect their social and cognitive development. Worrying about seizure was a significant subscale of the QOLIE-31 while medication effects had the least, thus, effective control of seizures frequency is essential to improve the HRQOL scores. The management of PWE should be aimed at both preventing seizures and reintegrating sufferers into community life. Public educational campaigns should be conducted in order to raise awareness of the public regarding the existence of effective therapy and eliminate the stigma of epilepsy. In conclusion, this study confirmed the findings of other studies regarding the importance of controlling seizures for a better QOL of PWE.

## Competing interests

The authors declare that they have no competing interests.

## Authors’ contributions

BN conceptualized the research, synthesized, analyzed and interpreted data and wrote the manuscript. JZ obtained the authorization permission, collected the data and partly wrote the initial draft of the manuscript. AK critically revised and edited the draft of the manuscript. All authors approved the final version of the manuscript. All authors read and approved the final manuscript.
